# Cholangiographic Features in the Diagnosis and Management of Obstructive Icteric Type Hepatocellular Carcinoma

**DOI:** 10.1155/2000/79241

**Published:** 2000-01

**Authors:** W. Y. Lau, C. K. Leow, K. L. Leung, Thomas W. T. Leung, Michael Chan, Simon C. H. Yu

**Affiliations:** 1 Department of Surgery The Chinese University of Hong Kong Prince of Wales Hospital Shatin Hong Kong; 2 Department of Oncology The Chinese University of Hong Kong Prince of Wales Hospital Shatin Hong Kong; 3 Department of Radiology The Chinese University of Hong Kong Prince of Wales Hospital Shatin Hong Kong

## Abstract

In 11 years and 3 months, 2037 patients with HCC were seen and 48 patients (2.4%) were diagnosed to have obstructive icteric type HCC. Five patients were terminally ill and were not investigated further. Forty three patients were initially investigated by endoscopic retrograde cholangiography (ERC) or percutaneous transhepatic cholangiogram (PTC) and classified as having obstructive icteric type 1, 2, or 3 HCC based on the cholangiographic findings. The obstruction in type 1 HCC was due to intraluminal tumour casts and/or tumour fragments obstructing the hepatic ductal confluence or common bile duct, while intraluminal blood clots, from haemobilia, filling the biliary tree was the cause in type 2 HCC. The pathology in type 3 HCC was extraluminal obstruction by extensive tumour encasement of the intra–hepatic biliary ductal system and/or extrinsic compression of the hepatic and common bile ducts by tumour(s) and/or malignant lymph nodes. At the initial ERC/PTC, 10 patients (5 resected, 50%) had obstructive icteric type 1 and 23 patients (0 resected) had obstructive icteric type 3 HCC. Of the 10 patients initially classified according to cholangiography to have obstructive icteric type 2 HCC, subsequent investigations revealed that 6 patients had type 1 HCC (4 resectable, 67%) and 4 patients had type 3 HCC (0 resectable). The classification of the obstructive icteric type HCC into types 1, 2, and 3, based on the initial cholangiographic appearances has simplified and rationalized our management strategy for this condition.


*HPB Surgery*, 2000, Vol. 11, pp. 299–306
Reprints available directly from the publisher
Photocopying permitted by license only

© 2000 OPA (Overseas Publishers Association) N.V.
Published by license under
the Harwood Academic Publishers imprint,
part of The Gordon and Breach Publishing Group.
Printed in Malaysia.

### Cholangiographic Features in the Diagnosis and Management of Obstructive Icteric Type Hepatocellular Carcinoma

W. Y. LAU[a,*], C. K. LEOW[a], K. L. LEUNG[a], THOMAS W. T. LEUNG[b], MICHAEL CHAN[c] and SIMON C. H. YU[c]

[a] *Department of Surgery,* [b] *Department of Oncology,*
[c] *Department of Radiology, The Chinese University of Hong Kong, Prince of Wales Hospital, Shatin, Hong Kong*

*(Received 5 January 1998; In final form 10 July 1998)*

**In 11 years and 3 months, 2037 patients with HCC were seen and 48 patients (2.4%) were diagnosed to have obstructive icteric type HCC. Five patients were terminally ill and were not investigated further. Forty three patients were initially investigated by endoscopic retrograde cholangiography (ERC) or percutaneous transhepatic cholangiogram (PTC) and classified as having obstructive icteric type 1, 2, or 3 HCC based on the cholangiographic findings. The obstruction in type 1 HCC was due to intraluminal tumour casts and/or tumour fragments obstructing the hepatic ductal confluence or common bile duct, while intraluminal blood clots, from haemobilia, filling the biliary tree was the cause in type 2 HCC. The pathology in type 3 HCC was extraluminal obstruction by extensive tumour encasement of the intra–hepatic biliary ductal system and/or extrinsic compression of the hepatic and common bile ducts by tumour(s) and/or malignant lymph nodes. At the initial ERC/PTC, 10 patients (5 resected, 50%) had obstructive icteric type 1 and 23 patients (0 resected) had obstructive icteric type 3 HCC. Of the 10 patients initially classified according to cholangiography to have obstructive icteric type 2 HCC, subsequent investigations revealed that 6 patients had type 1 HCC (4 resectable, 67%) and 4 patients had type 3 HCC (0 resectable). The classification of the obstructive icteric type HCC into types 1, 2, and 3, based on the initial cholangiographic appearances has simplified and rationalized our management strategy for this condition.**

*Keywords:* Cholangiography, hepatocellular carcinoma, obstructive jaundice

### INTRODUCTION

Jaundice as a presenting symptom of hepatocellular carcinoma (HCC) occurs in 5–44% of cases [1–4]. Its occurrence can be secondary to parenchymal insufficiency due to the underlying liver cirrhosis and/or parenchymal infiltration by the HCC, to obstruction of the biliary tract by intraluminal tumour cast/fragments/blood clots, to extraluminal compression of the bile ducts by the tumour or obstruction by enlarged malignant lymph nodes at the porta hepatis. The majority of these icteric patients has underlying parenchymal insufficiency and a percutaneous

*Corresponding author. Tel.: +(852) 2632 2626, Fax.: +(852) 2637 7974.

ultrasound scan will demonstrate an underlying non–dilated biliary ductal system. HCC presenting as obstructive jaundice is rare. The reported incidence varies from 0.7–11.7% [4–9]. Lin coined the term "icteric type" HCC for these cases and stressed their peculiarity which can give rise to diagnostic difficulty [8]. Despite remarkable improvements in the diagnostic tools available for investigating obstructive jaundice, not infrequently, these cases are still misdiagnosed as cholangiocarcinoma or choledocholithiasis. Prompt recognition of this type of HCC with relief of the obstruction either by stenting or resection can lead to extended survival with good palliation and occasionally cure [6, 7, 9–12]. Kuroyanagi and colleagues suggested the use of early cholangiography in evaluating these cases in order to determine the subsequent management plan [13]. While Lee *et al.* and Wu *et al.*, have described three main types of cholangiographic appearances in their studies of these patients with obstructive icteric type HCC, the proposed types of cholangiographic characteristics tend to overlap and thus blur management decisions [6, 10]. In our study of 48 patients, we present the cholangiographic features of obstructive icteric type HCC on the initial cholangiogram and based on these cholangiographic findings we have established a management algorithm which we have found to be useful in the diagnosis and management of these patients.

### MATERIALS AND METHODS

### Patients

Over the period May 1984 to August 1995, 48 patients with HCC, Jaundice and dilated intrahepatic bile ducts on ultrasonography were seen at the Joint Hepatoma Clinic at the Prince of Wales Hospital, Hong Kong. All the relevant patients' information at presentation and at subsequent follow–up visits were recorded in a specially designed clinical chart and subsequently entered into a computer database. Routine laboratory investigations included full blood count, liver and renal function tests, prothrombin time, activated thromboplastin time, hepatitis B serology and serum alpha–fetoprotein level. The diagnosis of HCC was based on histology and/or an elevated serum alpha–fetoprotein level of more than 500 ng/L in patients with space–occupying lesion(s) within the liver.

### Imaging

Endoscopic retrograde cholangiography (ERC) was attempted in all patients. In those patients where ERC was unsuccessful, percutaneous transhepatic cholangiogram (PTC) was performed. The initial direct cholangiograms (ERC or PTC) were reviewed by an interventional radiologist, an endoscopist and a surgeon and the patients were classified as having obstructive type 1, 2 or 3 HCC.

*Obstructive Icteric Type 1 HCC* Intraluminal Tumour Casts and/or Tumour Fragments.

These patients had intraluminal tumour growth along the hepatic duct and extending past the confluence of the right and left hepatic ducts into the common hepatic duct (CHD) causing partial or complete biliary obstruction. The appearance of the intraluminal tumour cast resembles that of a cork in the neck of a bottle (Fig. 1). In some cases, only intraluminal filling defects in the distal CBD (with or without an associated tumour cast) were seen. These filling defects were not unlike those seen in choledocholithiasis. However, the edges of these filling defects due to tumour fragments were irregular, softer and less well–defined than those due to stones (Fig. 2).

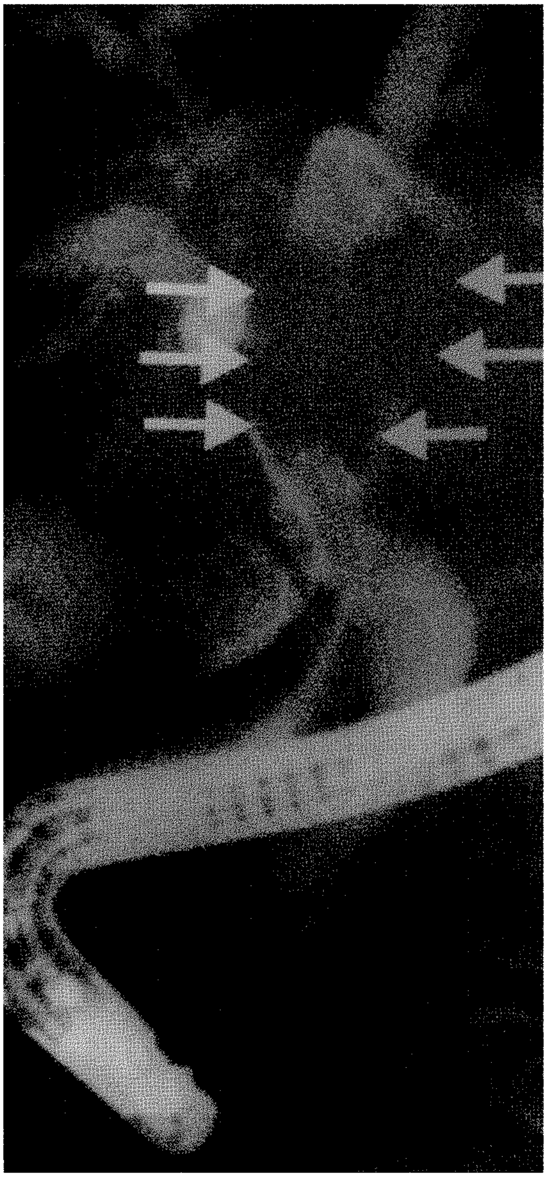

FIGURE 1 Obstructive icteric type 1 HCC – intraluminal obstruction by tumour cast. The appearance of the intraductal tumour cast resembles that of a cork within the neck of the bottle (arrows).

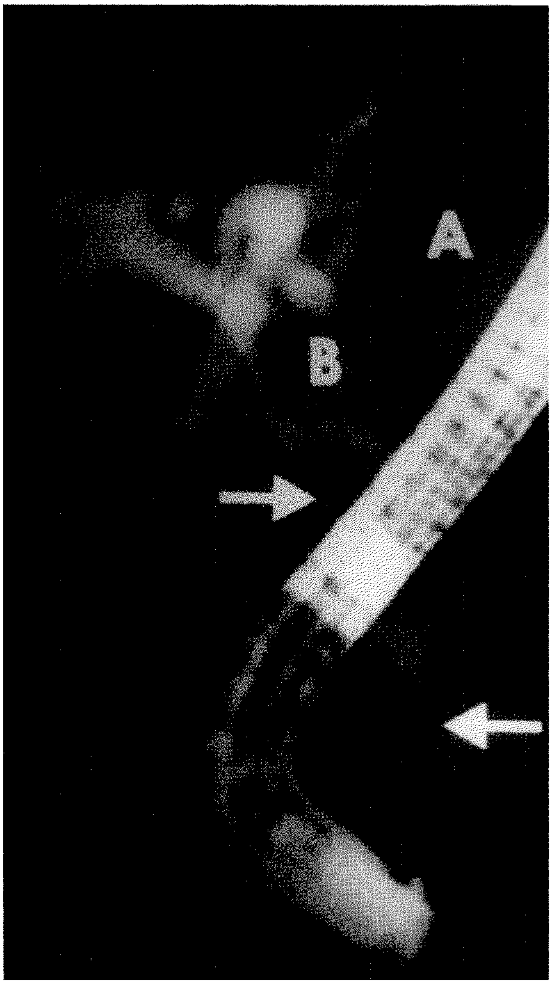

FIGURE 2 Obstructive icteric type 1 HCC – intraluminal obstruction by tumour cast and tumour fragments. ERC demonstrating the presence of tumour fragments (arrows), the main tumour (A) and the intraductal tumour cast (B).

***Obstructive Icteric Type 2 HCC*** Intraluminal Blood Clots Filling the Biliary Tree.

The whole of the CBD and hepatic ducts contained fluffy intraluminal filling defects due to blood clots secondary to haemobilia, thus obscuring the underlying lesion (Fig. 3). The appearance of the filling defects do not look as 'solid' as those due to tumour fragments.

***Obstructive Icteric Type 3 HCC*** Extraluminal Obstruction of CHD/CBD.

Tumour encasement and invasion of the hepatic ducts produced localised strictures and proximal

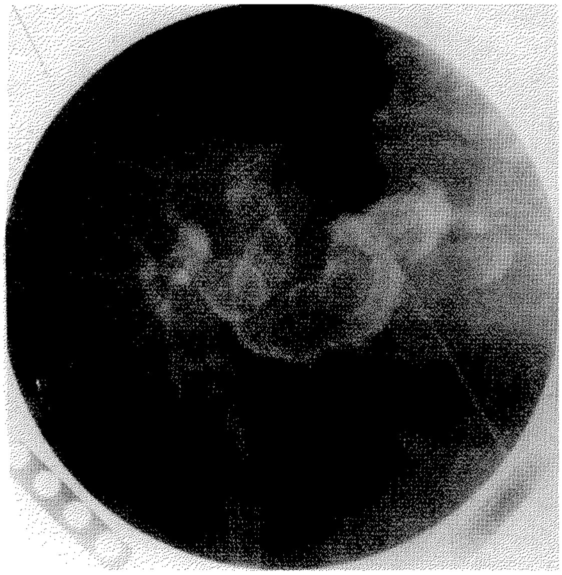

FIGURE 3 Obstructive icteric type 2 HCC – intraluminal obstruction by blood clots. The biliary tree is filled with intraluminal blood clots.

dilation of the ductal system. In addition, diffuse tumour involvement with stretching and displacement of the intrahepatic ducts in the whole liver were prominent features (Fig. 4). In some patients, the main obstruction was extrinsic compression of the CBD by enlarged lymph nodes at the porta hepatis (Fig. 5). However, this was also associated with an advanced liver tumour with diffuse involment of the biliary ductal system.

Patients with obstructive type 2 HCC had repeat ERC/PTC performed in order to delineate the exact nature of the underlying pathological lesion and to classify the patients into those having type 1 or type 3 HCC as the blood clot would have dispersed or moved. Those patients who were considered potentially operable underwent selective hepatic angiography (HA) and computed tomography (CT). Patients with inoperable HCC were palliated with endoscopic or percutaneous stents unless they were terminally ill. Those patients who have no evidence of bilobar disease and/or extrahepatic disease and/or thrombosed main portal vein were considered operable and underwent exploration. After thorough exploration and no intra–operative evidence of inoperability were found, the tumour was deemed resectable and completely excised.

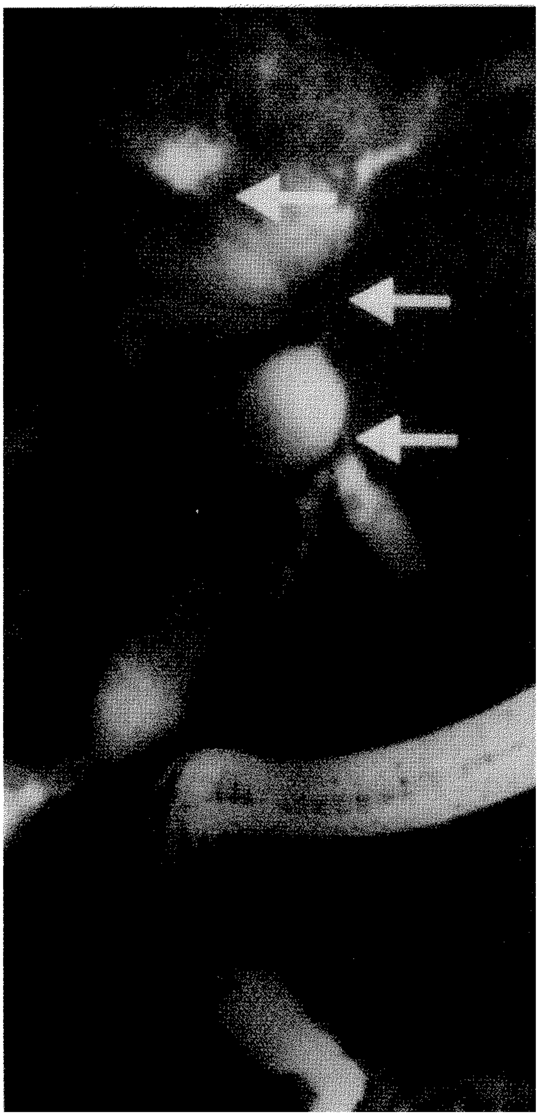

FIGURE 4 Obstructive icteric type 3 HCC – extraluminal obstruction. Tumour encasement and/or invasion of the hepatic ducts leading to localised strictures (arrows) and proximal dilatations.

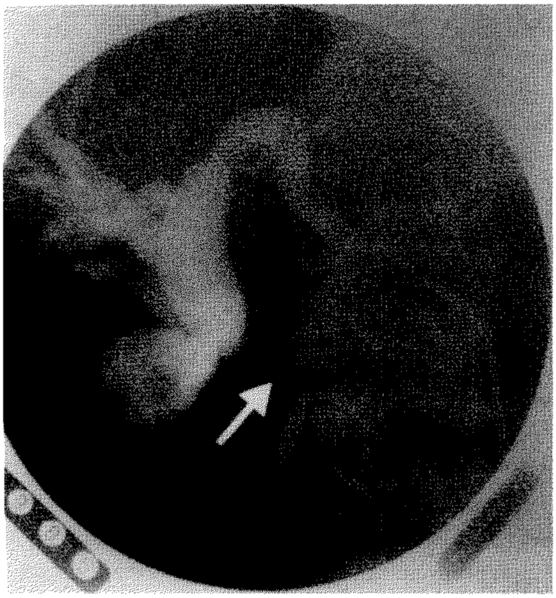

FIGURE 5 Obstructive icteric type 3 HCC – extraluminal obstruction. Extrinsic compression of the CBD by enlarged porta hepatis lymph node (arrow).

### RESULTS

In 11 years and 3 months, 2037 patients with HCC presented to the Joint Hepatoma Clinic. Only 48 patients (2.4%) had obstructive icteric type HCC. The mean age at presentation was 54 +/− 12.9 years and there were 39 males and 9 females.

All 48 patients had a dilated biliary system on ultrasound scan but only 43 patients (89.6%) had a liver tumour demonstrated on ultrasound. The liver function test revealed a mean total bilirubin level of 312.6 +/− 197.8 μmol/L (normal ≤ 15 μmol/L) and a mean alkaline phosphatase level of 402.9 +/− 250.1 μmol/L (normal ≤ 136 μmol/L). The hepatitis B surface antigen was present in 44 patients (91.7%). None were hepatitis C positive. Twenty six patients (54.2%) had a diagnostic alpha – fetoprotein level of over 500 ng/ml (range in 48 patients was 3–486,000 ng/ml).

ERC was attempted in 43 patients and the common bile duct (CBD) was cannulated successfully in 35 patients (72.9%). PTC was performed on the 8 patients who had failed ERC. No ERC or PTC was attempted in 5 terminally ill patients. After the initial ERC/PTC, 16 patients were further investigated for operability with computed tomography and hepatic angiography.

Five of the 10 patients (50%) with obstructive icteric type 1 HCC were found to be operable on subsequent investigations and the tumours were resected with 'curative' intent. Haemobilia in the 10 patients with obstructive icteric type 2 HCC settled spontaneously and subsequent ERC showed that 6 patients (60%) had obstructive icteric type 1 HCC and obstructive icteric type 3 HCC affected the other 4. Four of these 6 patients with obstructive icteric type 1 HCC were subsequently found to be operable. None of the 23 patients with obstructive icteric type 3 HCC had resectable disease.

One additional patient was explored surgically but the tumour was found not to be resectable and surgical intubation was performed. Twenty six patients were palliated with endoscopic stents and 5 with percutaneous stents. Technical difficulties in 2 patients necessitated the use of a combined percutaneous and endoscopic approach for the insertion of stents.

At five years, four of those patients treated surgically were alive. None of these patients treated medically with or without a stent survived 5 years (Fig. 6).

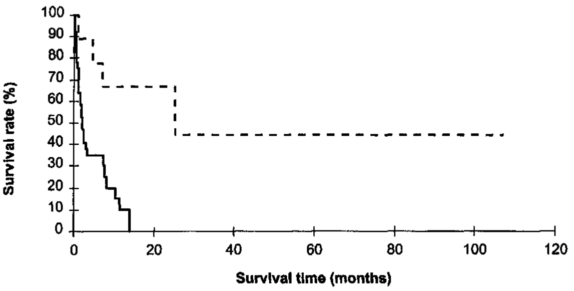

FIGURE 6 Survival curve of surgically treated (– – – – –) and medically treated (——) patients with obstructive icteric type HCC.

### DISCUSSION

The pathology, clinical course and outcome of the majority of these patients have been reported elsewhere [14]. This study deals specifically with the cholangiographic features found to be pertinent in the diagnosis and management of obstructive icteric type HCC at our institution.

The description of 3 main types of cholangiographic appearances by Lee *et al.*, emphasised the extrahepatic and intrahepatic appearances of the ductal system [6]. It is obvious from their description that overlap of the different types of appearances occurs within a single case, thus giving rise to ambigious information on the biliary tree which would affect management planning. In contrast, we place sole emphasis on the initial cholangiographic appearances of the main ductal system at the hilum in guiding us on our subsequent management. Obstructive jaundice due to an underlying HCC occurs only when the main biliary ductal system has been occluded by an intraluminal lesion or by an extraluminal compressive force. The former can occur in 3 ways: (1) after invading a branch of the intrahepatic ductal system, the tumour grows distally towards and into the CHD and the tumour cast obstructs biliary drainage from both liver lobes; (2) tumour fragments having separated from the proximal intraductal growth fall into and obstruct the distal CBD; and (3) haemorrhage from the tumour can partially or completely fill the biliary tree with blood clots. Equally, tumour encasement and/or extraluminal compression by an enlarged lymph node leading to obstructive jaundice will need to involve the main ductal system around the porta hepatis and not just the secondary ductal system.

Using the concept that intraluminal or extraluminal obstruction of the main biliary ducts causes obstructive jaundice in these patients with icteric type HCC, we have devised a management algorithm based on the initial cholangiographic features (Fig. 7). Nine out of the 16 patients (56%) diagnosed as having obstructive icteric type 1

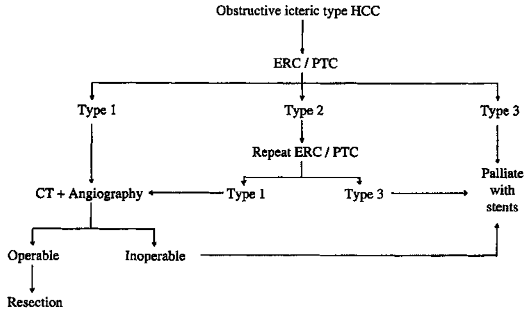

FIGURE 7 Management algorithm for obstructive icteric type HCC based on the initial cholangiographic features.

HCC on cholangiography were found to have resectable lesions (5 with tumour cast or "cork sign" and 4 with tumour fragments) at subsequent surgical exploration. The cholangiographic appearance of obstructive icteric type 2 HCC is indeterminate and is a consequence of haemobilia. Haemobilia secondary to haemorrhage from HCC may partially or completely fill the entire biliary tree with blood clots [9, 15–17]. It is difficult to differentiate between haemobilia due to HCC or to other causes on cholangiography alone. When haemobilia is diagnosed during ERC or PTC, it is advisable to establish biliary drainage, allow the haemobilia to subside and then repeat the cholangiogram to better assess the cause of the bleeding and determine whether it is type 1 or type 3 obstructive icteric type HCC. This strategy, in managing our 10 patients with obstructive icteric type 2 HCC, led to the identification of 6 patients with an underlying obstructive icteric type 1 HCC, of whom 4 underwent subsequent exploration and resection. The other 4 patients had icteric type 3 HCC. Thus, all patients with type 1 and 2 icteric type HCC should be investigated further and appropriate patients should be offered surgical exploration +/− resection.

Extraluminal obstruction of the biliary ductal system, as seen in obstructive icteric type 3 HCC, can be due to extrinsic compression by the underlying HCC and/or enlarged lymph nodes at the porta hepatis. In advanced HCC, tumour encasement of the biliary system leading to irregular strictures and proximal dilatations on cholangiography are not uncommon [6, 10] and can be confused with carcinoma of the bile duct. Differentiating obstructive icteric type HCC from cholangiocarcinoma can be difficult. On cholangiogram, bile duct carcinoma causes a "rat tail" stricture in 70% of the cases. In 25% a bulky tumour grows along the long axis of the duct and in 5% polypoid lesions within the duct will give rise to filling defects on cholangiography [18]. Although no one cholangiographic feature is diagnostic of HCC, the presence of the cholangiographic appearances as described here in a jaundiced patient with positive hepatitis B serology and/or cirrhosis would make the diagnosis of obstructive icteric type HCC most likely.

HCC is a rare condition in the North American and European continents. However, it is the most common cancer in the world and is probably the most common malignancy in males and is responsible for 1 million deaths per year [19, 20]. Obstructive icteric type HCC is rarer but prompt intervention in the appropriate patients in the form of palliative drainage of the obstructed system or surgical resection can lead to prolonged survival and potentially cure [6, 7, 9–13, 21–25]. We have found that the classification of the obstructive icteric type HCC into types 1, 2 and 3, based on the cholangiographic appearances described above, has simplified and rationalized our management strategy for this uncommon but potentially salvagable condition.

### *References*

[1] Edmonson, H. A. and Steiner, P. E. (1954). Primary carcinoma of the liver. A study of 100 cases among 48,900 necropsies. *Cancer*, **7**, 462–503.

[2] Kappel, D. A. and Miller, D. R. (1972). Primary hepatic carcinoma. A review of thirty-seven patients. *American Journal of Surgery*, **124**, 798–802.

[3] Kew, M. C. and Geddes, E. W. (1982). Hepatocellular carcinoma in rural Southern African Blacks. *Medicine*, **61**, 98–108.

[4] Ihde, D. C., Sherlock, P., Winawer, S. J. and Fortner, J. G. (1974). Clinical manifestations of hepatoma. A review of 6 years' experience at a cancer hospital. *American Journal of Medicine*, **56**, 83–91.

[5] Okuda, K. (1976). Clinical aspects of hepatocellular carcinoma–analysis of 134 cases. In: *Hepatocellular carcinoma*, Edited by Okuda, K. K., Peters, F. L., pp. 387–436. New York : John Wiley.

[6] Lee, N. W., Wong, K. P., Siu, K. F. and Wong, J. (1984). Cholangiography in hepatocellular carcinoma with obstructive jaundice. *Clinical Radiology*, **35**, 119–123.

[7] Kojiro, M., Kawabaata, K., Kawano, Y., Shirai, F., Takemoto, N. and Nakashima, T. (1982). Hepatocellular carcinoma presenting as intrabile duct tumor growth. A clinicopathologic study of 24 cases. *Cancer*, **49**, 2144–2147.

[8] Lin, T. Y. (1976). Tumors of the liver. Part I. Primary malignant tumors. In: *Gastroenterology*, Edited by Bockus, H. L., **3**, 3rd edn., 522–534. Philadelphia: WB Saunders Co.

[9] Lau, W. Y., Leung, J. W. C. and Li, A. K. C. (1990). Management of hepatocellular carcinoma presenting as obstructive jaundice. *American Journal of Surgery*, **160**, 280–282.

[10] Wu, C. S., Wu, S. S., Chen, P. C. *et al.* (1994). Cholangiography of icteric type hepatoma. *American Journal of Gastroenterology*, **89**, 774–777.

[11] Tsuzuki, T., Ogata, Y., Iida, S., Kasajima, M. and Takahashi, S. (1979). Hepatoma with obstructive jaundice due to the migration of a tumor mass in the biliary tract: Report of a successful resection. *Surgery*, **85**, 593–598.

[12] Roslyn, J. J., Kuchenbecker, S., Longmire, W. P., Tompkins, R. K. (1984). Floating tumor debris. A cause of intermittent biliary obstruction. *Archives of Surgery*, **119**, 1312–1315.

[13] Kuroyanagi, Y., Sawada, M., Hidemura, R., Aoki, S. and Kato, H. (1977). Common bile duct obstruction by hepatoma. *American Journal of Surgery*, **133**, 233–235.

[14] Lau, W. Y., Leung, K. L., Leung, T. W. T. *et al.* (1995). Obstructive jaundice secondary to hepatocellular carcinoma. *Surgical Oncology*, **4**, 303–308.

[15] Chen, M. F., Jan, Y. Y., Jeng, L. B., Hwang, T. L., Wang, C. S. and Chen, S. C. (1994). Obstructive jaundice secondary to ruptured hepatocellular carcinoma into the common bile duct. Surgical experience of 20 cases. *Cancer*, **73**, 1335–1340.

[16] Lai, S. T., Lam, K. T. and Lee, K. C. (1992). Biliary tract invasion and obstruction by hepatocellular carcinoma: report of five cases. *Postgraduate Medical Journal*, **68**, 961–963.

[17] Vaginos, C., Karavias, D., Dragotis, C., Kalofonos, H. and Androulakis, J. (1993). Obstructive jaundice due to intracholedochal blood clots: an unusual early presentation of primary hepatic carcinoma. *British Journal of Clinical Practice*, **47**, 222–223.

[18] Legge, D. A. and Carlson, H. C. (1972). Cholangiographic appearance of primary carcinoma of the bile ducts. *Radiology*, **102**, 259–266.

[19] Kew, M. C. (1992). Tumours of the liver. *Scandinavian Journal of Gastroenterology*., Supplement. **192**, 39–42.

[20] Rustgi, V. K. (1988). Epidemiology of hepatocellular carcinoma, pp. 390–391. In: *Hepatocellular carcinoma. NIH conference*, Di Bisceglie AM, moderator. *Annals of Internal Medicine*, **108**, 390–401.

[21] Afroudakis, A., Bhuta, S. M., Ranganath, K. A. and Kaplowitz, N. (1978). Obstructive jaundice caused by hepatocellular carcinoma. Report of three cases. *Digestive Diseases*, **23**, 609–617.

[22] vanSonnenberg, E. and Ferucci, J. T. (1979). Bile duct obstruction in hepatocellular carcinoma (Hepatoma)—Clinical and cholangiographic characteristics. Report of 6 cases and review of the literature. *Radiology*, **130**, 7–13.

[23] Brand, S. N., Brandt, L. J., Sprayregan, S., Brenner, S. and Bernstein, L. H. (1976). Extrahepatic biliary tract obstruction secondary to a hepatoma—containing blood clot in the common bile duct. *Digestive Diseases*, **21**, 905–909.

[24] Dickinson, S. J. and Santulli, T. V. (1962). Obstruction of common bile duct by hepatoma. *Surgery*, **52**, 800–802.

[25] Waldron, R. L., Kenny, G. and Sorger, K. (1973). Liver—cell carcinoma presenting as bile—duct tumour. *British Journal of Radiology*, **46**, 195–197.

